# The *Jewish religious heritage continuum*: Jewish religious communities’ interactions with synagogues and ceremonial objects in Amsterdam

**DOI:** 10.1080/13527258.2024.2334234

**Published:** 2024-04-09

**Authors:** Paul Ariese

**Affiliations:** Amsterdam School for Heritage, Memory and Material Culture, University of Amsterdam, and Reinwardt Academy, Amsterdam University of the Arts, Amsterdam, The Netherlands

**Keywords:** Synagogues, ceremonial objects, heritagisation, musealisation, Jewish museums, Jewish religious communities

## Abstract

This article explores how rabbis, directors and members of Amsterdam’s Jewish religious communities view the heritagisation of Jewish religious life by analysing how they interact with Amsterdam’s main synagogues and their collections of ceremonial objects. It focuses on the synagogues of the Jewish Cultural Quarter – the Portuguese Synagogue with its accompanying Sephardi community, and the former Ashkenazi synagogue complex, now the Jewish Museum. From a dynamic heritage perspective, this heterogeneous constellation raises questions about how and why heritage making occurs here. Following a Constructivist Grounded Theory methodology, concurrent data collection and analysis let emerge interrelated conceptual categories that explain how communities interact with these functioning and musealised synagogues and objects: *Embodying the transmission of tradition*; *Instrumentalising the heritage of Jewish religious life*; *Transforming the beauty of holiness*; and *Assembling in heritagised synagogues*. These categories intersect in the core category of the *Jewish religious heritage continuum*, which this article presents as a dynamic embodiment of remembering, reconnection, and revival of Jewish tradition. For the interviewees, these performances, and the deployment of functioning and musealised synagogues and collections, form a cultural apparatus that marks their present, diverse and living material culture and grafts a Jewish future onto a Jewish past.

## 1. Introduction

**Figure 1.**
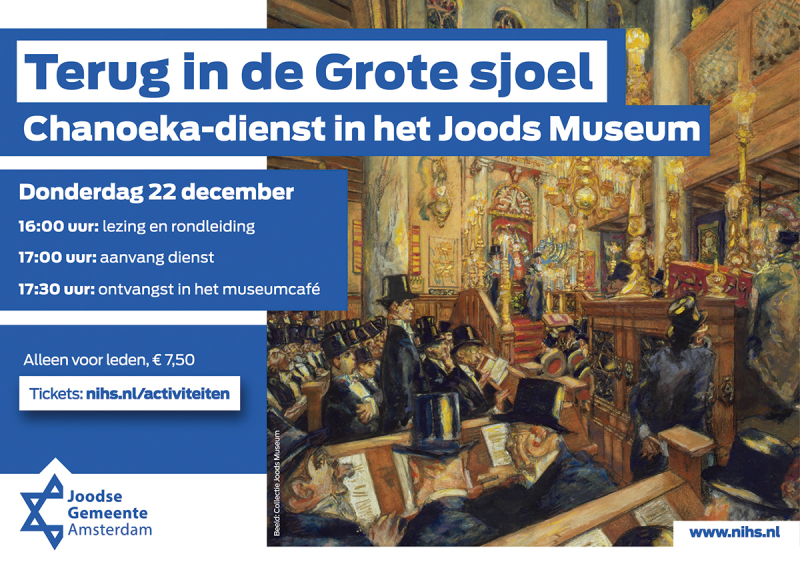
‘Back in the Great Shul’ flyer, NIHS (2022).

‘Back in the Great Shul. Hanukkah service at the Jewish Museum’ exclaims the flyer that Amsterdam’s Jewish Community, NIHS, mailed to its members in December 2022 (Figure 1). ‘For the first time in 80 years a Hanukkah service at this historic location!’ the NIHS website added (Joodse Gemeente Amsterdam 2022). The announcement is intriguing, since religion and heritage are not naturally compatible realms. Often, religious communities experience the identification of religious objects as ‘heritage’ as controversial, with its connotation of secularisation, desacralisation, and fossilisation (Alba 2022; Gross 2021a; Meyer 2023). In musealised or ‘multiple-use’ synagogues, the mix of religion and heritage may lead to confusion or a conflicting sense of ownership among managers, visitors, and other stakeholders (Heimann-Jelinek and Sulzenbacher 2022; Knufinke 2018). With this Hanukkah service, the NIHS returned to a place that despite an eight-decade hiatus still holds meaning to them. The Jewish Museum in the former Ashkenazi synagogue complex and the nearby Portuguese Synagogue, or ‘Snoge’, are part of Amsterdam’s Jewish Cultural Quarter.^1^ From a dynamic heritage perspective, this disparate ensemble raises some interesting questions: When does a religious site or object become heritage, and who decides? And what is the agency of synagogues and ceremonial objects defined as heritage (Frijhoff 2007; Ojeda-Mata and Schlör 2021)?

The present article discusses these issues by exploring how rabbis, directors and members of Amsterdam’s Jewish communities see the heritagisation of Jewish religious life, focusing on their interaction with Amsterdam’s main synagogues and the ceremonial objects kept there.^2^ By giving this seldom-heard group of heritage makers and users a voice, this study fills a gap in the discourse on the formation and interpretation of Jewish religious heritage (Ariese 2022; Paine 2019). A few exceptions are David Clark’s (2014) work on ‘performing community’ in Italian Jewish spaces and Pignatelli’s (2021) ethnographic study on the divergent involvement of official agents and local Jews in Jewish cultural heritage formation in Bragança, Portugal. Cyril Isnart (2023) applies the lens of the ‘religious heritage complex’ (Isnart and Cerezales 2020) to the treatment of Hebrew epigraphy at Tomar synagogue museum in Portugal, to illustrate how it serves simultaneously as tangible heritage object and ritual spiritual device. However, rather than describe the phenomenon examined here using predefined concepts, the present article explores the lived experience of people associated with Jewish communities and then develops a framework that explains in conceptual terms the interplay between embodied rituals, the spatial and material forms in and through which these people act, and their cultural stories and histories. Moreover, by recognising Jewish communities as ‘subjects’ rather than ‘objects’ of memory, the article espouses an increased awareness of the social significance of heritage (Martínez-Ariño 2020, 247; Pignatelli 2020).

## 2. Heritagisation and Jewish religious life

Before discussing the structure and results of this empirical study, I shall explain my approach to the interplay of people, spaces and objects in the heritagisation of Jewish religious life. The term ‘Jewish religious life’ rather than ‘Judaism’ indicates that this article focuses on the heritage process within a dynamic and diverse ‘family of traditions’ (Wright 2012, 17) and not on what is the heritage of a world religion or a fixed doctrinal framework. Using the term ‘heritagisation’ also positions the research. The term is associated with a critical approach to heritage as a discursive and social practice, developed by heritage and museum studies scholars such as Rodney Harrison (2013) and Laurajane Smith (2006, 2021). Heritagisation represents an ongoing ‘metacultural production’ or ‘cultural process’, shaped by the past, yet creating a future reality by integrating contemporary values and meanings (Harvey 2019, 22; Kirshenblatt-Gimblett 2014). In this approach, (religious) heritage is a contested construct because it is the provisional outcome of a process of inclusion and exclusion, motivated by interests and emotions. My goal in researching the heritagisation of synagogue space and ceremonial objects, is to provide insight into the mutual influence of ‘religious historical consciousness’ and the ‘pressure of contemporary heritage uses’ (Thouki 2022, 1055). Focusing on social interactions at these ‘theatres of memory and places of heritage-making’, rather than on the sets and props, reveals the motives behind this transformative performance: people create heritage, and use heritage to achieve recognition and self-affirmation, to connect with previous generations, to commemorate and accommodate experiences of loss, and to share what motivates and concerns them with others (Gross 2021b; Smith 2021, 2).

Heritage intervention – limiting use to protect ceremonial objects or opening synagogue spaces to museum visitors – affects the community’s experience. To understand the impact of heritagisation on religious materiality and practice, the where, when, and how of religious observance must also be studied from the community’s perspective, not just the museum’s. The embellishment of synagogues and ceremonial objects, and the enhancement of the beauty of religious rituals and festivals with decorative ornamentation stems from the Talmudic concept of *hiddur mitzvah*. This key concept, literally ‘beautifying the command’, is based on Exodus 15:2, ‘This my God and I will glorify Him’ (JPS). Art historian Batsheva Goldman-Ida explains that *hiddur mitzvah* is not so much about the visual beauty of Jewish places, objects and practices, but about what this beauty induces in the worshippers’ perception (2017). The question of the interpretation of ritual forms and practice links this article to the ongoing debate about the place and representation of religion in museums (Buggeln and Franco 2018; Franke and Matter 2022). In a museum setting, overlooking the origins of religious material culture can easily lead to misinterpretations of its aesthetic qualities. By contrast, exhibition spaces that represent and enable religion by embracing material practice ensure that religious objects need not lose their original significance (Berns 2015; Howes 2017). A holistic approach allows us to explore whether and to what extent synagogue spaces and ceremonial objects retain their agency in the entanglement of ‘religion as practice’ and ‘religion as heritage’ and provides insight into the uses of functioning and musealised synagogues as ‘cultural tools’ (Smith 2021, 27), despite their different nature and significance.

The following section introduces the synagogues in Amsterdam featured in this article. Constructivist Grounded Theory methodology is then explained as it pertains to this study. The accumulated data is presented in empirically grounded conceptual categories that converge in the *Jewish religious heritage continuum*. This set of interrelated concepts is elaborated in the Discussion section, in a dialogue with existing theory on the heritagisation of religion. In the course of the Discussion section, options for future research on the heritagisation of Jewish religious life are identified.

## 3. Amsterdam’s synagogues, a history of heritage

Six months before the NIHS flyer was released, I interviewed Shmuel Katz, an NIHS rabbi, at the Jewish Cultural Centre in Amsterdam Zuid. Rabbi Katz was talking about his plan to celebrate Hanukkah at the Jewish Museum. He spoke with passion when he said: ‘Finally, we’re back in town!’^3^ The location was no random choice. Opened in 1671, the Grote Sjoel (Great Shul) is part of the Ashkenazi synagogue complex in Amsterdam’s city centre, the heart of NIHS community life until the Second World War. Here the NIHS celebrated its tricentenary in 1935, even though attendance had declined in the previous decades due to secularisation and the move of many Jews away from the slums of Amsterdam’s city centre. The flyer proudly shows Monnickendam’s depiction: a packed Grote Sjoel, colourful *parochot* (ark curtains) lining the walls, and glittering ceremonial objects, including the magnificent Rintel Hanukkah candelabrum to lend extra cachet.^4^ That exhilarating moment lasted only briefly. In September 1943, when the Nazis declared Amsterdam ‘judenfrei’, they closed the complex. Soon after, the buildings were ransacked for anything of value or that could be used as fuel (Mendes de Costa 1944). Over 60,000 of Amsterdam’s Jews would never return. In 1954, an eviscerated NIHS sold the complex to the city. It was not until 1987 that it reopened as home to the Jewish Historical Museum. The event led historian Jaap Meijer, a Shoah survivor, to remark: ‘We – those Jews – actually no longer exist. Only our/their buildings are left, and they have now been given a useful purpose. They have been entombed!’ (Meijer 1987, 7). Today, the Grote Sjoel houses a permanent ‘Religion’ display.

For centuries, the Snoge, just like the Grote Sjoel, has attracted visitors and dignitaries from within and beyond the Jewish world (Kaplan 2015; Knotter 2004). However, few people entered the Snoge after the disruptive war years, except for the small Portuguese Jewish community. The reopening of the Ashkenazi synagogue complex as a museum gave the Snoge’s *parnassim* (directors) cause to reflect: ‘How can we revive this old place?’ Michaël Minco, who chaired the community’s board from 2017 to 2022, recalls.^5^ Unlike the Ashkenazi synagogue complex, the Snoge, which had first opened in 1675, survived the war almost intact, as did its collections. However, the Portuguese Jewish community had lost about 80% of its members, as had the far larger NIHS. Nevertheless, for the survivors and post-war generations, to give up their iconic Snoge, symbol of their mythical Portuguese past, seemed inconceivable.^6^ Even so, by the 1990s, the task of preserving the historical monument and its pristine seventeenth-century interior was causing severe financial strain, raising the spectre of the small Portuguese Jewish community having to sell some of its world-class ceremonial objects.^7^ In 1999, the *parnassim* commissioned curator Mirjam Knotter to compile an inventory and conserve the gradually deteriorating ceremonial collection. Knotter’s efforts raised awareness of the collection’s significance for the Portuguese community’s sense of identity.^8^ Meanwhile, cooperation with the Jewish Museum intensified.

Finding itself unable to raise sufficient funds to renovate the synagogue, and disqualified from government assistance as a religious community, a collaborative arrangement with the Jewish Historical Museum emerged as an increasingly attractive solution. The envisaged collaboration would enable the community to continue using the building for religious services and functions while the financial burden of renovation and upkeep would fall to the museum, which would also pay a substantial annual subsidy to the community for the use of the building. In 2003, the collection formally acquired protected heritage status under the Cultural Heritage Preservation Act and a foundation was set up – Portuguese Jewish Community Cultural Real Property Inheritance – through which the community would lease its historic real estate, including Beth Haim cemetery at Ouderkerk aan de Amstel. In addition, in 2007, the Portuguese Jewish Community Cultural Personal Property Inheritance foundation acquired legal ownership of the ceremonial objects and Ets Haim Library. The world’s oldest active Jewish library, housed at the Snoge since 1675, was registered under the UNESCO Memory of the World programme. While the community no longer owned its material heritage, it remained its principal user, explains Hans van Veggel, then chair of the umbrella Foundation for the Cultural Inheritance of the Portuguese Jewish Community (CEPIG).^9^ In 2009, management of CEPIG and care of the buildings and collections passed to the Jewish Museum.^10^ Everything that happens at the Snoge as part of Jewish Cultural Quarter is in line with its religious function. It is closed to the public on Shabbat and Jewish holidays, when its historic ceremonial objects are used in services. On other days, a museum ticket allows visitors access to the synagogue and its so-called Treasure Chambers, an open storage display created for the collection of ceremonial objects as part of the Snoge’s 2011–2012 restoration.

Although the opening of the Jewish Historical Museum in 1987 and the launch of the Jewish Cultural Quarter in 2012 can be seen as a reclamation of Jewish space in Amsterdam’s historic Jewish neighbourhood, Jewish religious life is far less evident in public than in the pre-war period. Today, most of the synagogues, Jewish schools and shops are found in southern parts of the city, in Zuid and Buitenveldert, and the adjacent municipality of Amstelveen. The main synagogue of the NIHS is Obrechtsjoel in Amsterdam Zuid – also known as Rav Aron Schuster Synagogue. Obrechtsjoel, the finest work of Jewish architect Harry Elte (Amsterdam 1880–Theresienstadt 1944) and considered the successor to the Grote Sjoel, opened in 1928. Hailed as an icon of Jewish emancipation and integration in the interwar period and of the reconstruction of Jewish life after the Shoah, the building was listed as a historical monument in 1996 (Van der Lans, Blocq, and Van Bergeijk 2023). Another landmark in what has become a diverse religious and cultural Jewish landscape is the synagogue, dedicated in 2010, of Amsterdam’s Liberal Jewish Community, numerically the largest in the Netherlands. Synagogues, traditionally described as ‘houses of assembly, learning and prayer’, may also be viewed as ‘houses of memory’ (Wallet 2017, 407). The synagogues discussed in this article embody critical moments and developments across four centuries of Jewish life in Amsterdam.^11^

## 4. Material and methods

How contemporary Jewish communities approach the entanglement of ‘religion as practice’ and ‘religion as heritage’ at Jewish museums, heritage sites and musealised synagogues in particular, is an insufficiently researched subject (Jimber Del Rio et al. 2020). Generating a conceptual framework to explain this relationship, rather than simply describe it, requires an open research approach. The iterative procedures of Constructivist Grounded Theory methodology have therefore been used in this study; a concurrent conducting, coding and analysing of interview data provides the basis for the development of conceptual categories and theory building (Charmaz 2014). Constructivist Grounded Theory adopts a relativist perspective, acknowledging the researcher’s input as a co-constructor of the data and the existence of varying perceptions of reality among interviewees (Birks and Mills 2023). Whenever it served the purpose of dialogue, I clarified my perspective as an outsider looking in at Judaism and explored with the interviewees the possible influence of my institutional affiliation as a lecturer in museum and heritage studies and my Protestant background on the interpretation of the subject discussed with the interviewees. These conversations led me to move away from my intellectualising approach and focus on religion as a material and sensory practice. I conducted interviews with three rabbis, three board members, and nine individuals with ties to Amsterdam’s main Jewish religious communities: the Portuguese Jewish Community ‘Kahal Kados Talmud Torah’, the Jewish Community of Amsterdam (referred to as NIHS: Nederlands Israëlitische Hoofdsynagoge), and the Liberal Jewish Community. For a perspective on Jewish communities outside Amsterdam, I interviewed Rabbi Hannah Nathans, former rabbi of Amsterdam’s independent Progressive Jewish congregation Beit Ha’Chidush, currently Open Jewish Congregation ‘Klal Yisrael’ in Delft, and Chief Rabbi Binyomin Jacobs of the Interprovincial Chief Rabbinate.

The seventeen interviewees, four women and thirteen men, range in age from early 20s to 80. The selection of rabbis and directors follows from their formal position within the respective communities. Other interviewees were selected with a view to the authority that people from this first group attributed to them, or through informal networks. The interviewees spoke from their affiliation to their Jewish community, as religious or cultural Jews, but also as Amsterdam residents and Dutch citizens. I also interviewed Hans van Veggel, CEPIG’s chair until 2021, and Mirjam Knotter, Chief Curator and Head of Exhibitions at the Jewish Cultural Quarter. The semi-structured interviews lasted between 45 and 105 minutes. Almost all were conducted face-to-face. Some took place at the Snoge, other locations included the Jewish Cultural Centre of the NIHS, Obrechtsjoel, the Liberal synagogue or at the interviewee’s home. Interviews at the Snoge and Obrechtsjoel were ‘walking interviews’, allowing for more insight into the interviewee’s experience of space and the memories evoked here (Hunt and D’Errico 2022, 216). Following historian Judy Jaffe-Schagen, all interviewing was conceived as a time-specific construction of connections between objects and places, interviewee and interviewer (Jaffe-Schagen 2013). Topics covered in each interview were the interviewees’ understanding of the term ‘Jewish religious heritage’, their interactions with synagogue space and ceremonial collections in a religious or heritage context, and personal ties to the Jewish Cultural Quarter synagogues and collections. Furthermore, implications of opening the Grote Sjoel and the Snoge to a broader, non-Jewish and/or non-religious audience were also discussed.

## 5. Results

Comparing the interviewees’ – often personal – stories, I identified patterns and connections in the conditions that influenced them, their actions and emotions in these circumstances, and their consequences (Urquhart 2023). The Results section explains this interplay in five conceptual categories. The first is *Embodying the transmission of tradition*. The other categories are *Instrumentalising the heritage of Jewish religious life*; *Transforming the beauty of holiness*; and *Assembling in heritagised synagogues*. Building on these, this section concludes with the core category, the *Jewish religious heritage continuum*.

### 5.1. Embodying the transmission of tradition

The interviewees’ perception and uses of synagogues and ceremonial objects belong to a time and place continuum. Interviewees, both Liberal and Orthodox, agreed that for Jewish life to flourish, they need to recognise their roots. It became evident to me that this recognition is not just an idea, but a basic attitude and an embodied and living practice. *Embodying the transmission of tradition* is not confined to heritage institutions but first becomes tangible in the context of lived religion. The transmission of Jewish tradition depends on constant repetition in the present, explains Ruben Vis, the Amsterdam based secretary of the umbrella organisation of the Orthodox Jewish Communities in the Netherlands: ‘Remembering is interwoven with our daily prayer’.^12^ His explanation echoes Itizk Peleg’s understanding of Jewish tradition as a constant ‘remembering, telling and identifying’ with the generations of the past (Itzik Peleg 2022, 119). A phrase often repeated in Scripture and Jewish liturgy is *le-dor-va-dor*, meaning from generation to generation. Boaz Cahn, a secular university student, considers the intergenerational transmission of tradition essential for the Jewish people to survive. He epitomises this principle through his active participation in the educational work of the Liberal community.^13^ Assimilation and migration, and minority status in a sometimes blatantly and otherwise latently hostile environment can disrupt this transmission, the Shoah being the ultimate caesura: ‘It’s a miracle that we’re still here, and I hope we’ll stay… That continuity is therefore fundamental’, notes Rabbi Nathans.^14^

The Snoge, with its magnificent seventeenth-century interior and its disturbing emptiness, reflects the impact of the Shoah and of the post-war process of rebuilding Jewish life, migration, and secularisation. The building also symbolises ties with Portuguese Jewish communities and synagogues elsewhere. While ‘Kahal Kados Talmud Torah’ member and former chair of the *parnassim* David Cohen Paraira takes me through the Snoge, he reveals how intergenerational and supralocal connections become tangible in the transmission of objects: The *tebah* (the central reading platform, equivalent to the Ashkenazi *bimah* referred elsewhere) and some of the chandeliers and benches in the Snoge originate from one of the earlier seventeenth-century Portuguese synagogues in Amsterdam. Ets Haim’s former study room in the peripheral buildings was converted into a small winter synagogue during the Snoge’s 1954–1959 restoration. Its *hekhal* (the holy ark, where the Torah scrolls are kept, equivalent to the Ashkenazi *aron hakodesh* referred to elsewhere) comes from one of the pre-war Portuguese Jewish nursing homes, while its *tebah* was newly made in matching style to fit the sacred cloths from the Portuguese Synagogue in The Hague, of which only a few members survived the Shoah.^15^ Similar connections are visible in Amsterdam’s Liberal synagogue: The idea of *le-dor va-dor* motivated Rabbi Ten Brink to reinstall the eternal light, which he explains as the symbol of the continuity of Jewish life since the time of the Temple in Jerusalem, from the previous shul: ‘Every active and living Jewish community has a *ner tamid*. That the late Frieda Menco, an Auschwitz survivor, relit this eternal light at our synagogue’s dedication made it even more symbolic’.^16^

**Figure 2.**
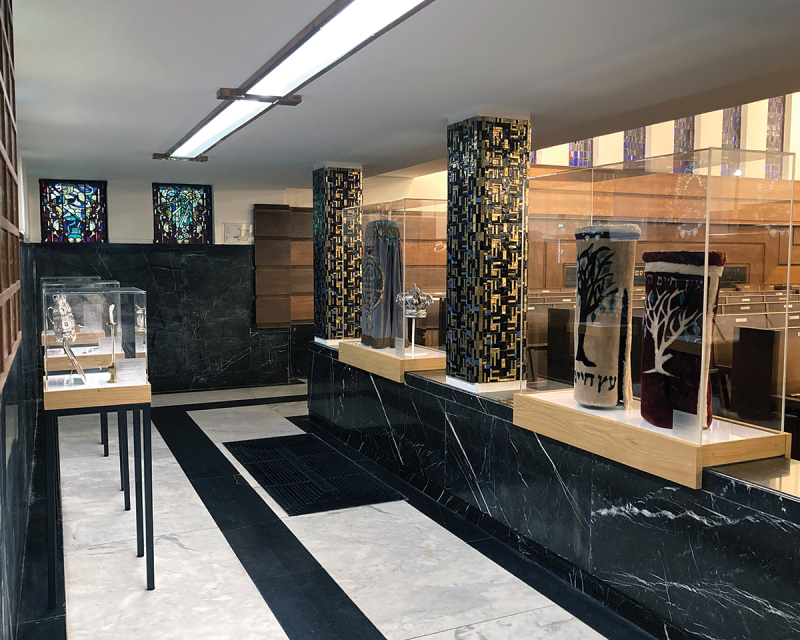
Ceremonial objects on display at Obrechtsjoel. Photo: Paul Ariese, 2023.

Board member Paula Blocq demonstrates a similarly people-centred approach to heritage as she shows me around her Orthodox Obrechtsjoel in Amsterdam Zuid. The oak benches and six stained-glass windows depicting the twelve tribes and the Jewish holidays on either side, give the black-and-white Art Deco interior a warm atmosphere. On entering the shul space, I am welcomed by a series of showcases (Figure 2) containing the blue velvet Torah mantle donated by the board of the Jewish Community to mark the shul’s inauguration in 1928. Blocq, who installed these showcases in 2018, employing Rietbroek Oudijn Designers, a long-time Jewish Museum partner, explains that the objects are too fragile to use regularly yet too important not to show. The community’s pride is a copy of the silver Rintel Hanukkah candelabrum at the Jewish Museum.^17^ The lighting of this historic showpiece attracts families from the entire NIHS. Blocq’s commitment has enabled the community to embrace the building and its collection as ‘living heritage’. She concludes that her goal has been achieved: ‘Making that heritage visible has strengthened the mutual bond’.^18^ The 2017–2021 restoration of the synagogue encouraged the small community to open up to people from outside, with lecture programmes and guided tours.^19^

**Figure 3.**
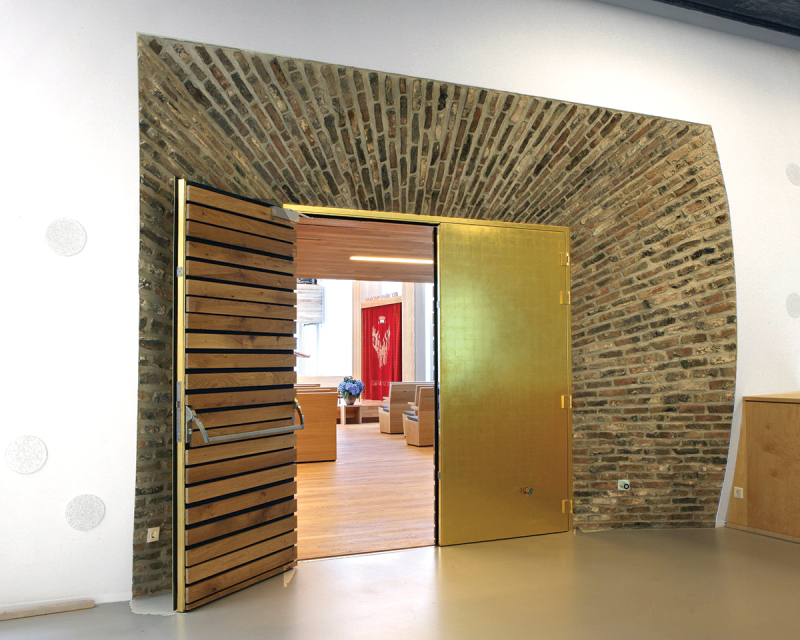
Entrance to the Liberal Jewish Community synagogue. Photo: Amsterdam City Archives, Doriann Kransberg.

*Embodying the transmission of tradition* is also about taking responsibility within and beyond the synagogue. Many interviewees see themselves as part of a larger whole, connecting past and future. When asked why he agreed to serve as chair of the *parnassim*, Minco explains that his family have been part of the Snoge since the seventeenth century: ‘I feel emotionally connected to this place. I’m not a religious person, but I feel that I enjoy contributing as a volunteer’.^20^ Rabbi Katz refers to his own nineteenth-century Gerard Dou shul as he explains his desire to pass on his predecessors’ legacy: ‘My chair in the synagogue, that’s where Chief Rabbi J. Tal sat after the war; that’s an enormous responsibility’.^21^ Rabbi Ten Brink of the Liberal Jewish Community aptly illustrates the notion of connecting generations, recalling how he urged architect Bjarne Mastenbroek (seARCH) to reuse bricks from the community’s former synagogue (1966–2007) in the present building (Figure 3). The former synagogue’s architects interpreted these bricks, burned rejects from the factory, as a metaphor for the Shoah victims whose bodies were burned in the camp ovens, and the traumatised survivors. To Rabbi Ten Brink, the new synagogue’s construction embodies the continuity of Judaism: ‘We are a people, and we are proud that we can pass that on, always, each in their own time, in their own way’.^22^

*Embodying the transmission of tradition* shows how synagogues, *klei kodesh* and mere functional objects materialise the idea of continuing Jewish life and space. During the interviews and site visits, I noticed that the tradition of preserving the religious function of objects once dedicated for worship is motivated by more than just practical considerations. Their reuse, like the ritual performance itself, helps keep previous generations present. Synagogues and their collections form not just material but, as it turns out, also social assemblages. Here, conservation, exhibition and reuse are primarily intracultural operations through which communities join their past, present and future. However, applying formal heritage frameworks to Jewish religious materiality means that, in addition to the perpetuation of ‘social memory’, its significance in terms of ‘cultural memory’ also increases. While the former concerns participation in intragenerational transmission, the latter focuses on the cultural forms through which the engagement with the past is mediated, including by those without personal involvement in these histories (Macdonald 2013, 15). As the following section reveals, this expansion arouses diverse emotions and tensions.

### 5.2. Instrumentalising the heritage of Jewish religious life

Whereas the interviewees aspire to preserve and transmit their tradition, they also face external and internal threats to their community. This situation is all but new: as early as the late nineteenth century, assimilation, secularisation and demographic changes in Jewish communities brought about a reconsideration of the meaning of Jewish religious material cultures, leading to the establishment of scholarly societies focused on collecting, preserving and presenting Judaica and Jewish museums in major cities in West and Central Europe, including Amsterdam (Berger 2018; Heimann-Jelinek and Schmid 2022). This development was reinforced by ambivalent relationships with the non-Jewish environment, the ‘othering’ of Jews, and the recurring question of what it means to be Jewish. Contemporary heritagisation of Jewish religious life, either originating in Jewish communities themselves or in institutions such as the Jewish Cultural Quarter, aims to ensure the survival of these communities in a diverse society. In this context, religious buildings and objects function as *pars pro toto* for a tradition and a people under pressure. By labelling its materiality as heritage, communities perpetuate a religious or cultural sense of Jewishness. Moreover, communities use these buildings and objects within practices of representation to address the non-Jewish, non-religious environment.

Heritagisation inevitably influences how interviewees experience religious practice. Sam Herman is the former assistant of successive rabbis of the Portuguese Jewish community, *shamash* and *mashgiach* (beadle and kashrut supervisor). In this capacity, he came to appreciate that partnering with the Jewish Museum is the only way to preserve the Snoge for the community, yet he also sees drawbacks. Commenting on my use of the concept of heritagisation, he responded: ‘Heritagisation represents a devaluation of religious content in favour of spectacle, compromise, and mimicked authenticity. From a religious perspective, the museum is a corrupting influence and the community’s association with the museum must eventually lead to the abandonment of orthodoxy.’ Herman emphasises that heritagisation brings unrest to the surface that has roots in internal tensions, such as the community’s increasing diversity versus a desire among some for uniformity.^23^ Heritage interventions often take place at the tipping point between time-transcending traditions and contemporary local realities, within and around communities. However, in addition to forms of heritagisation developed *within* communities, this also implies a heritagisation *of* communities. The interviewees’ response to this role shift shows that different motives underlie their shared pursuit of continuity.

The Shoah interrupted the oral transmission of religious tradition, forcing post-war generations to reconstruct a religious past and practice from fragmentary memories. Hence, the primary motive is to compensate for knowledge that was not passed down. ‘People wanted to restore the memory of a Jewish world that no longer exists’, Bar Vingerling, the office manager of the Portuguese Jewish community, notes. ‘They had to fall back on older written sources instead of a dynamic, oral tradition’.^24^ When heritage intervention challenges this invented tradition – in some respects, all that remains – people may become emotional, resisting change to avoid imminent loss.^25^ Attempting to restore tradition is in itself a loss, Rabbi Nathans observes: ‘If you try to reconstruct it all with your head, you lose the heart of things’.^26^

The materialisation of identity is another motive for the use of synagogues and ceremonial objects as cultural tools. At the Snoge, Rabbi Abraham Rosenberg recalls a discussion with some *snogeiros* (Snoge members) about a fragile Torah mantle: ‘They believed: It’s donated to be used and you should use it as long as possible. To which I say bluntly: “Listen, would you let your wife go to a party in a torn, worn-out dress?”’^27^ For these *snogeiros*, the historical collection’s symbolic affirmation of their Portuguese Jewish identity prevents them adopting a more conciliatory attitude and respecting museological restrictions in ritual practice. For them, the collection is vital: possessing the objects means possessing the tradition. By contrast, heritage intervention in the NIHS collection has posed less of a challenge to identity. Vis notes: ‘There’s a consistent lack of interest in heritage preservation here, in Jewish heritage. Objects are simply seen as utensils’.^28^ For people whose approach to ceremonial objects is purely functional, it’s puzzling to have to consider their fragility when discussing their use – that is nothing short of a paradigm shift.

Clinging to the familiar is a third motive: Vingerling and Ruben Troostwijk – the latter as chair of NIHS’s board – notice that people in their communities feel uncomfortable or even annoyed when required to perform rituals with objects other than those they normally use. For many congregants, objects determine their festive experience.^29^ To accommodate these sensitivities, the Jewish Cultural Quarter and the *parnassim* have drawn up a detailed protocol for the responsible use of objects in the Snoge, drawing on archival research and oral tradition. The guiding principles are (i) preservation of tradition; (ii) conservation of fragile objects, limiting or ending their active use if susceptible to irreversible damage; (iii) decorum: ending use of damaged objects; (iv) remembrance of donors; and (v) available staff (Knotter et al. 2015). On festivals, the silver-gilt Augsburg *kohanim* basins and ewers are displayed at the *hekhal*, so they contribute at least visually to the service (Knotter 2013, 164). Cohen Paraira recognises that this greater caution has not yet led to a greater appreciation among fellow *snogeiros* for the collection’s heritage value or material value, so that some valuable items end up damaged in the depot.^30^

Finally, reassessing the ritual also plays a role in the pursuit of continuity: Rabbi Rosenberg and Vingerling attribute the tension that heritagisation brings to a one-sided focus on ritual form. For them, it’s the rationale behind the ritual that matters.^31^ For others, however, the aesthetics of ritual play a major role in their bond with the Snoge. Interviewee KKTT#1’s roots are in the Ashkenazi community. His father once brought him to the Snoge on Tisha be-Av, the annual day of mourning for the destruction of the Jerusalem Temples. ‘They’re so cheerfully sad there’, he said. The experience of the synagogue in which everything shiny was covered with black cloth has remained with him: ‘The cultural heritage is what I want to preserve. I think that tradition, in essence, is a lot of nonsense, but I think it’s beautiful’.^32^

The heritagisation *of* communities tends to emphasise tangible and time-specific traces of the development of Jewish religious life in interaction with the local, non-Jewish environment. In contrast, heritagisation *within* communities – the immaterial culture of using, reusing, remembering, and reviving – emphasises the transtemporal and supralocal kinship with other Jews. The key to understanding Jewish communities’ religious modes and motives as they deal with the heritagisation of their religious lives is a relational approach, shifting attention from the condition of space and objects to the interplay between their use and users. Meanwhile, the fragile situation in which Jewish communities find themselves makes the question of the social and performative dimensions of musealised synagogues and objects even more pressing. What remains when these dimensions transform or recede is discussed next.

### 5.3. Transforming the beauty of holiness

Synagogues, objects, and rituals, such as the Torah reading, enable the worshipper to fulfil the *mitzvot* (commandments). Through ritual performance, a ceremonial object becomes holy. The splendour of objects and rituals make that transition to holiness a conscious act. However, when Jewish communities initiate or enable heritage intervention, they enter an ambiguous world. The heritagisation of a synagogue or ceremonial object implies that layers of meaning suddenly or gradually start to shift, with a risk of upsetting the balance between beauty and holiness. When buildings and objects are valued, preserved, and presented solely for their beauty, they become distant, and indefinably empty. What is going on in the figurative void surrounding ‘authorised heritage’? For some interviewees *Transforming the beauty of holiness* prompts a reappraisal of what might otherwise have been lost. Others experience this transformation as a devaluation or loss of meaning. ‘The experience of Judaism is a continuum’, Vis emphasises. ‘Suddenly, someone says: “This is a museum piece … ” Well, that causes serious tension!’^33^ For Rabbi Jacobs, a mezuzah on a Jewish doorpost gives residents a material focus for devotion. But shown in a museum, that same object can be a metaphor for the murder of those residents and the decline of Jewish religious observance.^34^

**Figure 4.**
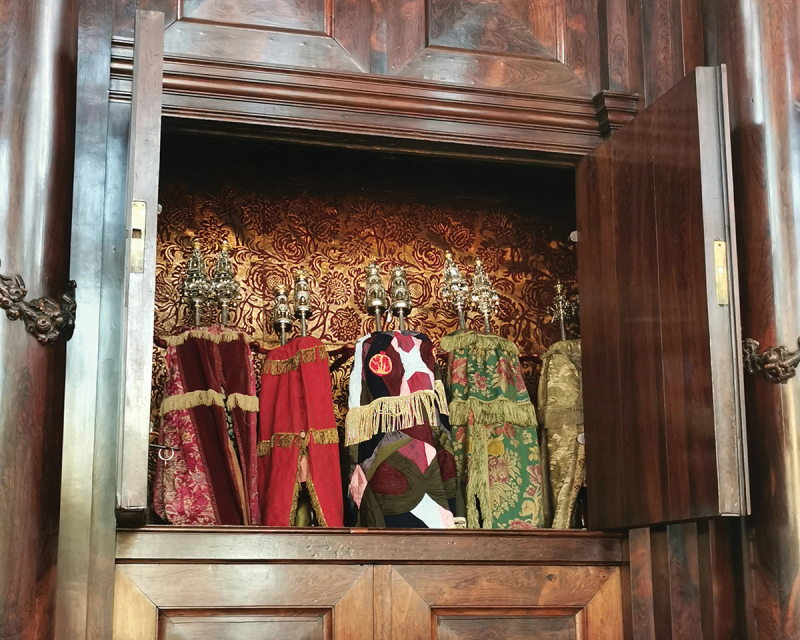
Mattijs van Bergen’s Torah mantle flanked by centuries-old ceremonial objects in the Snoge’s *hekhal*. Photo: Jewish Cultural Quarter, 2022.

These remarks reveal how Jewish museums may portray a moribund religion, quite different from the living Jewish community the interviewees want to see represented.^35^ When asked how the Snoge differs from the musealised Grote Sjoel, Minco emphasises: ‘The Snoge is a place of life. Actually, all synagogues are a place of life; they are not supposed to be musealised, petrified, put in a display case’. A striking example of ‘being a living community and not just a museum’, he argues, is the joint Portuguese Jewish community, CEPIG and the Jewish Cultural Quarter initiative to commission couturier-artist Mattijs van Bergen to design a new Torah mantle (Figure 4), to mark the retirement of CEPIG’s first chair, Hans van Veggel.^36^ Also for interviewee LJG#2, a member of the Liberal community, Jewish heritage is vibrant: ‘“Heritage”, if you’re Jewish, is never finished, it’s not a fossil, you can’t encapsulate it!’^37^ She shows me her carefully documented collection of hundreds of Jewish prayer books. Most of them are of little interest to a museum, but she preserves these well-thumbed *siddurim* as tangible testimonies to the lives of ordinary people. While objects may no longer be perceived as merely functional, the transformation process clearly affects how many of the interviewees position themselves as links in the ‘chain of generations’.^38^

More than mere religious utensils, the ‘living objects’ in Obrechtsjoel perpetuate the community’s memories, stories, traditions, and values.^39^ Comparing this in situ display to the ‘Religion’ display, I note a fundamental difference: at Obrechtsjoel, objects represent their makers and users, whereas in the museum they represent themes of Jewish religious life or illustrate a particular period in Jewish history. The idea of a link to individuals, or other times or places shifts irrevocably to the background, even though the labels explain that some objects have been at the Grote Sjoel for centuries. Nonetheless, exhibiting can also increase community awareness of the bigger picture, Rabbi Rosenberg observes: ‘Our heritage helps us understand that we’re just a cog in the machine’.^40^

I asked Cohen Paraira whether heritagisation affects the uses and meanings of ceremonial objects as we descend from the Snoge’s quiet courtyard to the Treasure Chambers, where the synagogal collection is kept in an open storage display. Here, amid the narrow vaults of the outbuildings, we return to the origins of the Portuguese Jewish community. ‘This is our community’s oldest object’, explains Cohen Paraira, with evident pride. ‘Our first rabbi Uri Halevi donated this Torah scroll in the early seventeenth century before he returned to Emden’.^41^ We look at a fragile parchment, dated *c*. 1400, covered by a deep red textile with floral and foliate motifs, on top of which lies a silver *yad* (pointer). All this rests on a centuries-old cloth embellished with delicate embroidery. Now that Uri Halevi’s scroll is permanently behind glass, I wonder whether the display emphasises the ‘holiness of beauty’ rather than the ‘beauty of holiness’ (The Rabbi Sacks Legacy n.d..). Cohen Paraira emphasises the scroll’s multiple significance: ‘While it now serves an educational purpose, it retains the same sacred status as before’.^42^ The attribution of heritage value has also led to a reconsideration of the scroll’s lifespan, argues Minco: ‘Why continue using precious objects until they fall apart, as some members suggest? At least now we can continue to view it!’^43^

When I revisit the Snoge a few months later, Rabbi Rosenberg recalls that, as Ets Haim library’s curator, he was the first to exhibit the scroll. ‘This community has a long history of displaying ceremonial objects’, he explains. ‘That shouldn’t be an issue at all’.^44^ Before the ceremonial collection’s transfer to CEPIG, Cohen Paraira was involved in its management and repaired objects that were damaged through use. Staff at the Jewish Museum have taken over the responsibilities of congregants, and the scroll is no longer there to be read, but to represent the community’s roots and resilience. Meanwhile, Herman points to the risk of perceiving the Snoge as a time-capsule of the premodern era. Incongruous post-war objects have been removed or sent to the small Snoge in Amstelveen, while ‘a surreal olde worlde atmosphere’ is fostered in Amsterdam.^45^

Unlike in 1987, when the ‘Religion’ display in the Grote Sjoel had a distinctly abstract design to stress that it would never again serve as a synagogue, the current display evokes the pre-war synagogue interior. The Torah scroll on the reconstructed *bimah* (the reading platform, equivalent to the Sephardi *tebah* referred to above) in the centre is less of an identity marker than Uri Halevi’s scroll. Once owned by Mozes Polak (1898–1965), chair of Middelburg’s Jewish community, it was donated to the Jewish Museum in 1984. The scroll is opened at the priestly blessing – ‘May the Lord bless you and protect you!’ – although the promise of God’s care remains unnoticed among visitors unfamiliar with Hebrew. Also not explained is that this Torah scroll is *pasul* (imperfect, therefore unfit for reading in a service).^46^ Normally, when a scroll cannot be restored, it is buried or stored away in a *geniza*, a repository for unusable Jewish ritual objects, especially those that contain the name of God (Schleicher 2010). Because the Torah is intended for learning, respectful display of a *pasul* scroll is halachically permissible, unlike a *kosher* scroll (Abelson 1997). For many interviewees, the Torah scroll is clearly being used for an educational, and thus legitimate, purpose here.^47^

In the course of this research, it dawns on me that for the interviewees the real issue when it comes to transforming and musealising Jewish religious material culture is not the *display* of sacred objects but the *invisibility* of their users and the *discontinuity* of their use. While the objects on display leave most of the interviewees relatively unresponsive, the centuries-long use of the Snoge and the Grote Sjoel as a house of assembly, learning and prayer clearly resonates for them. Referring to the museum and the nearby National Holocaust Names Memorial, interviewees repeatedly emphasised that Jewish life did not end with the Shoah and that the present-day Jewish community is a *living* community. Therefore, they expect the Jewish Cultural Quarter to represent the diversity of contemporary Jewish life, more than try to unravel what Judaism means. I argue that the latter also defines why, in the heritagisation process, the interviewees’ attention shifts from objects they can no longer use to places where they can continue to meet.

### 5.4. Assembling in heritagised synagogues

In a study of Jewish spatial practices in Barcelona, sociologist of religion Martínez-Ariño presents the concept of ‘place-recovering strategies’: Jewish communities and organisations seek recognition of their presence due to their historical and contemporary role as a minority group. These strategies include heritage production, place-making and place-marking as a way to reaffirm a Jewish presence at sites where this has previously been lost or is currently vulnerable (Martínez-Ariño 2020). A striking aspect of the creation of the Amsterdam Jewish Cultural Quarter is its use of historic religious space as a vehicle for the explication of a contemporary, but not necessarily religious Jewish presence. *Assembling in heritagised synagogues* explains why the synagogues of the Jewish Cultural Quarter, reclaimed Jewish space in the heart of Amsterdam’s historic Jewish district, leave none of the interviewees unmoved.

While the Grote Sjoel plays a role in the wider Amsterdam Jewish community’s collective memory, only a few of the interviewees have any personal memories of the synagogue.^48^ Asked about the ‘Religion’ display, the interviewees share feelings of sadness, alienation, and pride. The marble *aron hakodesh* (holy ark, like the Snoge’s *hekhal*), the only element that survived the war more or less unscathed, now displays Torah mantles, *rimonim* (finials) and crowns. The silver Rintel Hanukkah candelabrum stands where it previously stood and the *ner tamid* is reinstalled, although unlit. Nevertheless, for LJG#1, affiliated with the Liberal community, the exhibition design suggests ‘all the objects required for a shul to function are present’. Indeed, his embodied knowledge translates into a sensory performance: ‘I’m one of the people who read the Torah in my own shul. When I visit the museum with friends, I read from the Torah text displayed here to demonstrate the chanting’.^49^ For Cahn, however, the shiny ceremonial objects in the showcases convey a sense of distance, being used to the austere surroundings of his own shul.^50^ Rabbi Nathans, aware of the Grote Sjoel’s historicity, feels no real emotional connection: ‘The Jewish Museum doesn’t feel like a shul anymore; it’s just things’.^51^ Rather than the visual similarity or the exhibits, it’s the awareness that people gathered and prayed here for centuries that appeals to the imagination, Rabbi Katz, Troostwijk and Cahn confirm.^52^ LJG#2’s performance is profoundly influenced by this idea: ‘When I visit the Grote Sjoel, I say to myself [here she starts singing]: *Ma tovu*…, “How fair are your tents, O Jacob, Your dwelling, O Israel!” This is where it all happened. And, for me, it still happens here’.^53^ Several respondents emphasise the Grote Sjoel’s social dimension, characterising it as a ‘non-religious house of assembly’^54^ or ‘a Jewish house where guests from all over the world, and all denominations, and secular people’ meet.^55^

**Figure 5.**
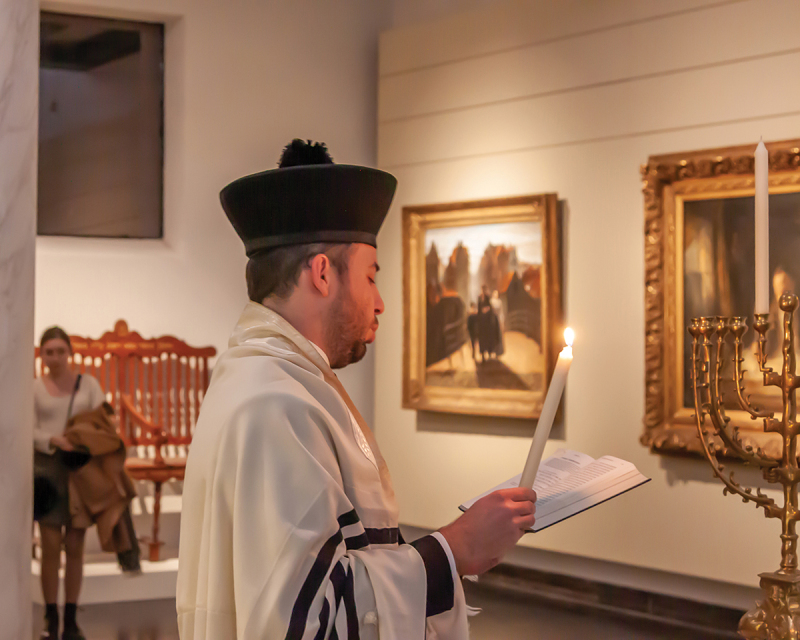
Hanukkah in the Grote Sjoel, December 22, 2022. Photo: Dirk P.H. Spits/DPHOTO.

Regarding their feelings towards the synagogues of the Jewish Cultural Quarter, interviewees express a sense of belonging, yet not unequivocally. Cohen Paraira rarely attends Snoge services due to his age and the distance from home – he doesn’t drive or use public transport on Shabbat. Yet on weekdays he can often be found as a volunteer in the Snoge or Jewish Museum, among the objects with which he feels familiar.^56^ LJG#1 values the museum as a symbol of Jewish Amsterdam’s revival^57^; but for Rabbi Jacobs it represents the decline of Jewish religious life: ‘It’s a soulless body now’, he concludes when asked about his perception of the Great Sjoel. ‘Once a shul has become a museum, there’s nothing Jewish about it. Though it’s better they turned it into a museum than a launderette’.^58^ In 1987, NIHS’s rabbinate boycotted the Jewish Museum since it opened on Shabbat.^59^ However, in recent decades, the Jewish Cultural Quarter has built trust through its programming and respectful handling of the various communities’ heritage.^60^ That the museum is now considered far less controversial is evident from the current rabbinate’s initiative to celebrate Hanukkah here (Figure 5).^61^ As Chazzan Sacha van Ravenswade filled the space with Hanukkah melodies, the people in between the showcases seemed to merge with the footage of those singing here in 1935, at the Jewish Community’s tercentenary, projected on the museum walls.^62^ The live singing – unheard here since the war years – transformed the space, revealing for a moment the temporality of the current exhibition.

Regarding the Snoge, interviewees share memories of coming together and feelings of belonging. Rabbi Rosenberg is one of those who recall the Yom Kippur service, as hundreds of people fill the candlelit space: ‘I’m very much a non-mystical person, but on that night, you feel the previous generations looking over your shoulder’.^63^ The age-old interplay between space and liturgy at the Snoge forms a bridge to ‘come home’, says LJG#2, who converted to Judaism later in life. ‘I realised there’s a tradition that’s alive and will stay alive. If I can connect with that, then I’ll be where I want to be’.^64^ The Snoge services create a precious and simultaneously vulnerable moment for its members. Museum staff maintain a delicate balance, facilitating the services without interfering in community life. However, the service is not experienced unequivocally. For Herman it’s a shadow of what it once was: ‘A theatrical Portuguese show learned by a few dedicated actors continues to be performed in antiquated evening dress, to the exclusion of all other participants, to create a superficial reconstruction replete with top hat, *ngayin* and staged *gravitas*’.^65^ By contrast, assembling in the Snoge puts Vingerling in a reflective mood. He recalls how, after a Friday evening service which only a few attend, he walks home and ponders: ‘This is Amsterdam’s best-kept secret. Everyone is partying, while here in Snoge, we’re singing melodies passed on for centuries […] Let the museum have the building; we have the service’.^66^

The heritagised synagogues evoke identification in some interviewees, even though these places were never their own. Meanwhile, they alienate others who hesitate to return to what was once familiar. Balancing history and memory, curated authenticity, and community dynamics, the Jewish Cultural Quarter strives to let religious heritage retain, regain, and renew meaning. While the objects that make up the ‘Religion’ display primarily originate in the Orthodox tradition, the exhibition emphasises a Liberal focus on *tikkun olam*, ‘working towards a better world’. The management provides space for living religion, as evidenced by the NIHS’s Hanukkah celebration. Nevertheless, following their institutional agenda of ‘making the Jewish story accessible to as much of the general public as possible’, they position the Jewish Museum primarily as a place to question rather than affirm what it means to be Jewish in today’s super-diverse and post-secular society.^67^ That is why I do not consider the museum a dead end; like the other synagogues in this article, it forms a multi-layered ‘infrastructure’ (Abakelia 2021, 323). This infrastructure mediates, through its interplay of the interviewees’ performance, spatial experience and narrative, the continuation of Jewish life in Amsterdam.

### 5.5. The Jewish religious heritage continuum

**Figure 6.**
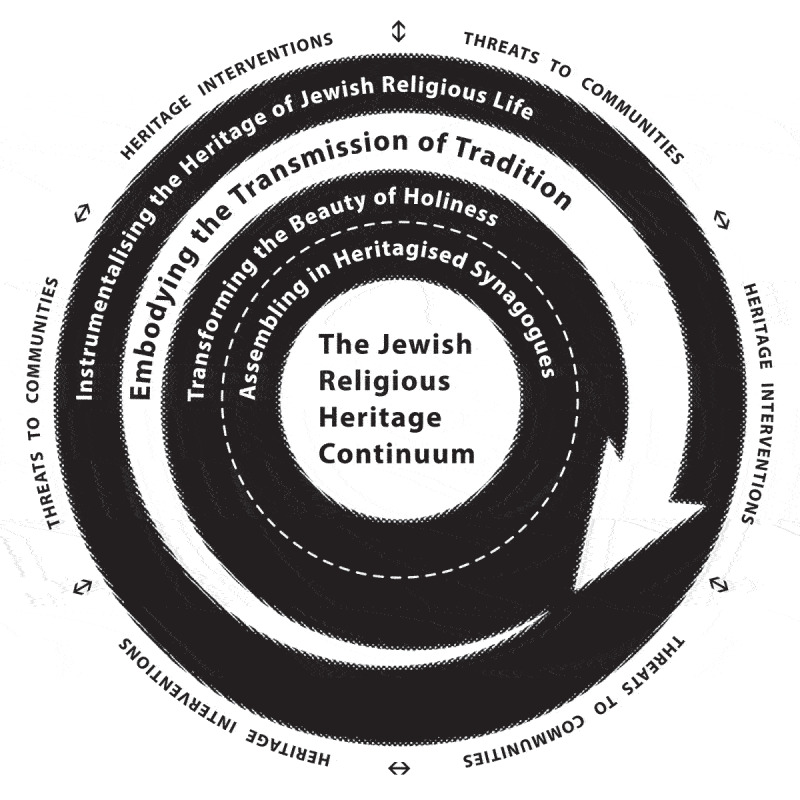
The *Jewish religious heritage continuum*. Paul Ariese.

The conceptual building blocks of the *Jewish religious heritage continuum* (Figure 6) reflect the interviewees’ desire to embody and materialise their Jewish experience expressed in a profound, diverse and enduring performance. Spaces and objects thus mediate intergenerational connections in the religious community, but in the family circle too. Merav Krone, a student with family ties to the Snoge but not a shulgoer herself, explains this as ‘living in the midst of things that help me shape my Jewish world’.^68^ *Embodying the transmission of tradition*, recalling the concept of *le-dor va-dor*, is central to the way interviewees maintain the connection with previous generations. Just as the tradition of *hiddur mitzvah*, the commandment to embellish the ritual act, is ultimately about those who carry out the commandment and not about the beautiful objects with which they do this, for interviewees in this article the synagogues and ceremonial collections in and outside the Jewish Cultural Quarter mediate the transmission of Jewish memory, place and identity. In contemporary, post-secular and diverse society, the Grote Sjoel, the Snoge, and their ceremonial collections join their in situ preserved and exhibited pendants as reference points, in ‘producing a continuity in a distinctive Jewish sensorium’ (Auslander 2017, 845). These findings lead me to interpret these buildings and objects not as a residue or relic of the past but as part and parcel of a living and evolving Jewish material culture.

The treatment of ‘Jewishness materialised’, a transforming, and constantly contested assemblage (Feldt and Zuckerman 2021, 13), also shows how boundaries with the non-Jewish environment are demarcated. In that sense, heritage interventions must be interpreted as both a cause and a consequence of internal and external threats to the Jewish community. Community responses to the resulting adjustments in ritual practice, categorised as *Instrumentalising the heritage of Jewish religious life*, range from resistance to accommodation or renewal. When religious materiality is classified as ‘heritage’ it generates and mediates new ritual forms and practices, while also triggering mechanisms aimed at preserving or commemorating a reinvented past. These responses operate at the intersection of fear of losing and striving not to let go. When changing and challenging circumstances in and around Jewish communities lead to heritage intervention, the consequence is *Transforming the beauty of holiness*. This transformation implies that the interviewees consciously reconsider notions of beauty and holiness, whereby the perpetuation of Jewish life remains paramount. *Assembling in heritagised synagogues* is an outcome of transforming heritage interventions. As this category shows, for some the synagogues become what memory studies scholar Alison Landsberg describes as ‘transferential spaces’, where people who lack a historical or family connection evoke ‘prosthetic memories’ (2004, 135–36). Other interviewees fill these spaces with new ritual performances, adding layers of meaning to what none of them experiences as a pure vacuum. In summary, the *Jewish religious heritage continuum* reveals how interviewees anticipate the changes and challenges of the present by living and reviving their heritage, grafting a Jewish future onto a Jewish past.

## 6. Discussion

The heritagisation of religion has moved up the agenda in museum and heritage studies over the past decade (Buggeln, Paine, and Plate 2017; De Jong and Mapril 2023; Franke and Jelinek-Menke 2022; Isnart and Cerezales 2020; Weir and Wijnia 2023). Ariese’s (2022) review of heritage studies, Jewish studies, and material religion literature reveals that the heritagisation of Jewish religious life is primarily explained with reference to the development and design of Jewish museums or exhibitions or the interpretation of specific collections. Curators of the Amsterdam’s Jewish Museum, Cohen (2013) and Van Voolen (2014), for example, have pointed out how synagogues and Jewish museums have the same goal of keeping Jewish memory alive, even though musealised synagogues or Jewish museums as a space for religious materiality are assumed to diverge from functioning synagogues serving as a place for religious time. The *Jewish religious heritage continuum* challenges this assumption, showing that people affiliated with contemporary Jewish communities combine community-specific practices of preserving and passing on religious traditions and materiality with external heritage initiatives. Moreover, the *Jewish religious heritage continuum* shows how meaning-making practices in interacting spatial, tangible, sensory and temporal dimensions in both functioning and musealized synagogues (Ariese 2022, 251) express a transtemporal way of transmitting Jewish tradition: By connecting past and future generations, by shaping their own place in the present, by using spaces and objects as images and counter-images in practices of representation, the community members fulfil the *mitzvot* to remember, to re-enact and to relive.

This article presents a multi-layered survey of what Jewish communities consider sacred when religion and heritage become intertwined. As witnesses to historical caesuras in the *Jewish religious heritage continuum*, museum spaces eventually acquire transcendent meaning and become sacred secular spaces for those involved in these histories, as Holocaust studies scholar Avril Alba also argues (2015). None of the interviewees envisage a restoration of the original use of the Jewish Cultural Quarter. What matters to them is that the spotlight is turned towards the heritagised as a performer in the *Jewish religious heritage continuum*, bringing meaning to the museum void similar to the meaning in the synagogue, at home, and elsewhere. As Jewish communities position themselves as actors involved in making Jewish heritage, rather than passive, distant source communities, Jewish museums are increasingly becoming living spaces (Meijer-van Mensch, Franke, and Jelinek-Menke 2022). Such a reciprocal relationship lends museums and their collections a polyphonic character. At the same time, this constellation enjoins participants to listen to each other with attentively, precisely because of the differences in tone of voice.

For a correct interpretation of the significance of Jewish religious heritage in and for today’s society, follow-up research should explore visitors’ perceptions of the Jewish Cultural Quarter, including unaffiliated Jews who far outnumber those involved in religious communities. This brings Erica Lehrer’s notion of ‘communities of implication’, which she proposes as an alternative to the detached notion of ‘source communities’ (Lehrer 2020), into play. One question is, for example, how museum audiences and Jewish heritage tourists do recognise the Amsterdam and Dutch Jewish communities’ ongoing involvement in the Jewish Cultural Quarter. And to what extent do the Jewish Cultural Quarter synagogues play a role in confirming or challenging visitors’ own (non) religious identification? How museum audiences interpret Jewish religious life is partly determined by how heritage professionals conceptualise this. Follow-up research should also focus on unravelling the approach of heritage professionals who work in and for this institution. Following Moody’s argument to consider the ‘history of heritage’ when assessing contemporary examples (Moody 2015, 125), the Jewish Cultural Quarter’s current approach should be explored against the backdrop of decades of collection development and exhibiting.

This research has revealed how Jewish religious communities consider synagogues and ceremonial objects as tangible traces of the past and eloquent witnesses in the present. Ultimately, the interviewees embody the story, forming a *Jewish religious heritage continuum* that encompasses secular and sacred functions. They show that, despite or perhaps because of the ambiguous position of its synagogues and ceremonial collections, the Jewish Cultural Quarter has the potential to address the historical and contemporary presence of the community and personal quests for what unites them in a diverse world in a positive light. As a house of assembly for visitors of all faiths and none, the Jewish Cultural Quarter offers a counter-narrative to that ever-present threat to essentialise and then engulf the Jewish people. This condition recalls the continuum’s origin: As the Israelites cross the divided Jordan, the last obstacle on their way into the Promised Land, their leader Joshua commands them to take twelve stones from the middle of the dry riverbed. At the next camp, the twelve tribe leaders pile up this collection. Earlier, Joshua had marked the place of origin, in the middle of the now raging river, with another twelve stones: ‘This shall serve as a symbol among you: in time to come, when your children ask, “What is the meaning of these stones for you?” you shall tell them […]’ (Joshua 4: 6–7). Whoever reads further will discover many more stories to tell and listen to, materialised in the stones of the Amsterdam synagogues.

## Notes

1. The Jewish Cultural Quarter was launched in 2012. Besides the Jewish Museum and the Portuguese Synagogue, the Jewish Cultural Quarter includes Jewish Museum junior, the National Holocaust Museum and the National Holocaust Memorial at Hollandsche Schouwburg and Ets Haim Library. In 2021, the Jewish Historical Museum was renamed ‘Jewish Museum’. The Portuguese Synagogue is still used by the Portuguese community as a place of worship and is known as the Snoge, from the Spanish *esnoga*.

2. In this article, ceremonial objects are: *klei kodesh*, objects that are sacred because they bear the name of God and *tashmishei kedushah*, which are sacred for being in close contact with *klei kodesh*; *tashmishei mitzvah*, key ritual tools, and *reshut*, objects that only serve to beautify the ritual act (Auslander 2017, 839; Heimann-Jelinek and Schmid 2022, 39–40).

3. Interview with Rabbi Shmuel Katz, Jewish Cultural Centre, Amsterdam. June 16, 2022.

4. Synagogue service commemorating the Ashkenazi Community’s tercentenary, November 14, 1935. Martin Monnickendam, 1935. Acquired with support of the Jewish Cultural Quarter Foundation. Collection Jewish Museum, M003250. The candelabrum is named after Sarah Rintel, who donated the object to the Grote Sjoel in 1753. In 2017, the NIHS sold the ‘Rintel’ to the museum. Collection Jewish Museum, M014776.

5. Interview with Michael Minco, interviewee’s office, April 6, 2022.

6. Interview with Rabbi Abraham Rosenberg, Portuguese Synagogue, Amsterdam. November 1, 2022.

7. Interview with David Cohen Paraira, Portuguese Synagogue, Amsterdam. May 11, 2022; Interview with Hans van Veggel, interviewee’s home. March 20, 2023; Interview with Sam Herman, Portuguese Synagogue, Amsterdam. June 8, 2023.

8. Interview with Mirjam Knotter, via Microsoft Teams. October 1, 2020; Minco.

9. Van Veggel.

10. The Torah scrolls are excepted; they remain the property and responsibility of the Portuguese Jewish Community

11. The standard work by Blom et al. (2017) provides a comprehensive survey of Dutch Jewish history with background to the developments referred briefly to in this section.

12. Interview with Ruben Vis, Jewish Cultural Centre, Amsterdam. April 7, 2022.

13. Interview with Boaz Cahn, Reinwardt Academy, Amsterdam. February 27, 2023.

14. Interview with Rabbi Hannah Nathans, interviewee’s home. November 2, 2022.

15. Cohen Paraira.

16. Interview with Rabbi Menno ten Brink, Liberal Jewish Community synagogue, Amsterdam. May 12, 2022.

17. The copy (1898) was made for the Dritt Sjoel, which, like the Grote Sjoel, is part of the Ashkenazi synagogue complex, now the Jewish Museum.

18. Interview with Paula Blocq, Obrechtsjoel, Amsterdam. March 9, 2023; Interview with Paula Blocq, via telephone. March 28, 2023.

19. Each year, the Obrechtsjoel welcomes hundreds of visitors on Heritage Day; Museum Het Schip, which showcases 1920s Amsterdam School architecture, organises monthly tours.

20. Minco.

21. Katz. The Gerard Dou shul (1891) is one of the few purpose-built synagogues in nineteenth-century Amsterdam. Coenen Snyder (2012) discusses the development of synagogues in that period in detail.

22. Ten Brink.

23. Herman.

24. Interview with Bar Vingerling, Portuguese Synagogue, Amsterdam. November 28, 2022.

25. Nathans; Rosenberg.

26. Nathans.

27. Rosenberg.

28. Vis.

29. Vingerling; Interview with Ruben Troostwijk, Jewish Cultural Centre, Amsterdam. March 20, 2023.

30. Cohen Paraira.

31. Rosenberg; Vingerling.

32. Interview, KKTT#1, interviewee’s home. May 27, 2021.

33. Vis.

34. Interview with Rabbi Binyomin Jacobs, interviewee’s home. March 27, 2023.

35. Ten Brink; Cahn; Minco.

36. Minco.

37. Interview with LJG#2, interviewee’s home. February 7, 2023.

38. Vis; Ten Brink; Minco; Katz; Vingerling; LJG#2; Cahn.

39. Blocq.

40. Rosenberg.

41. The Portuguese Jewish refugees, who had lived as Catholic *conversos* – compelled to convert, yet often secretly adhering to their Jewish identity as so called crypto-Jews – on the Iberian Peninsula for several generations before coming to Amsterdam, invited the Ashkenazi rabbi Moses Uri Halevi (*c*.1544–1627) of Emden to serve as their spiritual leader in 1601.

42. Cohen Paraira.

43. Minco.

44. Rosenberg.

45. Herman.

46. Rabbi Jacobs inspected the scroll [collection JHM M002298] in 1998 and declared it *pasul*. E-mail conversation, Henrike Hövelman, Head of Collections and Knowledge, Jewish Cultural Quarter. September 27, 2021. When visiting the Israel Museum in Jerusalem (June 2022), I noted that rabbinic advice had also been sought regarding the display of a *pasul* scroll in the ‘Jewish Art and Life’ galleries.

47. Ten Brink; Interview with LJG#1, Reinwardt Academy, Amsterdam. March 30, 2022; LJG#2; Nathans; Rosenberg; Troostwijk.

48. Troostwijk recalls that in addition to the *brit milah* (circumcision) of his son, a Jewish marriage also took place here in 2005.

49. LJG#1.

50. Cahn.

51. Nathans.

52. Cahn; Katz, Troostwijk.

53. The prayer said upon entering a functioning shul (Numbers 24:5); LJG#2.

54. Minco.

55. LJG#1.

56. Cohen Paraira.

57. LJG#1.

58. Jacobs.

59. Chief Rabbi Meir Just accused the museum of allowing Jewish Enlightenment ideals to prevail over religious principles (Just 1987a, 1987b).

60. Minco; KKTT#1; Troostwijk.

61. Troostwijk.

62. My thanks to Rabbi Katz for allowing me to attend the Hanukkah celebration at his personal invitation.

63. Rosenberg.

64. LJG#2.

65. Herman.

66. Vingerling.

67. Examples are exhibitions such as *Are Jews White?* (2021) which examines where Jews stand within the current spectrum of identity and representation politics and *Me, Jewish?* (2023), in which a diverse group of Dutch Jews show how they deal with (their) Jewish identity.

68. Interview with Merav Krone, Portuguese Synagogue, Amsterdam. May 16, 2023.
